# Adherence to post-surgery follow-up assessment and its association with sociodemographic and disease characteristics in patients with breast cancer in Central China

**DOI:** 10.1186/s12885-020-07600-y

**Published:** 2020-11-12

**Authors:** Ran Feng, Jingfeng Jing, Xiaojun Zhang, Ming Li, Jinnan Gao

**Affiliations:** 1 Department of Breast Surgery, The Affiliated Shanxi Bethune Hospital of Shanxi Medical University, 99 Longcheng Street, Taiyuan, 030032 Xiaodian District China; 2 Cancer Research Institute, University of South Australia, Adelaide, 5000 Australia

**Keywords:** Follow-up, Adherence, Breast cancer, China

## Abstract

**Background:**

Follow-up after curative surgery is increasingly recognized as an important component of breast cancer care. Although current guideline regulates the follow-ups, there are no relevant studies on the adherence to it in China. This study investigated the post-surgery follow-up and explored its association with patients, tumor and treatment characteristics.

**Methods:**

A total of 711 patients underwent surgical treatment in Shanxi Bethune Hospital from March 2012 to May 2018 were included in this study. Baseline sociodemographic, tumor, and treatment characteristics were obtained from the hospital electronic medical records. The post-surgery follow-up was reviewed and assessed from the patient’s follow-up examination record. Factors associated with the first three-year follow up was evaluated using logistic regression analysis.

**Results:**

The annual follow-up rate after surgery decreased gradually from 67.1% at the 1st year, 60.2% at the 3rd year to 51.9% at the 4th year, and 43.5% at the 5th year. Loss of follow-up during the first 3 years after surgery was significantly associated with older age (> 65 years), lower medical insurance coverage, axillary lymph node dissection, and less intensity of systemic treatment.

**Conclusion:**

A significant downtrend of annual follow-up rate for breast cancer survivors was confirmed in this study. Loss of follow-up within the first 3 years after surgery was associated with both patient’s characteristics and treatment. These results will provide evidence to help clinicians to develop tailored patient management after curative surgery.

**Supplementary Information:**

The online version contains supplementary material available at 10.1186/s12885-020-07600-y.

## Background

The incidence of breast cancer has increased more than 30% in the past decades in China [1]. Epidemiological studies based on international and Chinese data have showed that breast cancer is the most common one in female malignant tumors [2, 3]. Breast cancer patients with early breast cancer have better survival, lower recurrence and metastasis rate due to early diagnosis and improved treatment [4].

As an important element in the comprehensive management of breast cancer patients [5], follow-up (hereinafter as FU) can eliminate treatment-related complications, detect recurrence and metastasis as early as possible, and improve mental health and quality of life. The National Comprehensive Cancer Network (NCCN) clinical practice guidelines suggest monitoring breast cancer patients closely and recommending mammography every 12 month [5]. Chinese guidelines recommend that a FU every 3 months in the first 2 years after the operation (or after adjuvant chemotherapy is finished), every 4 to 6 months for the 3rd to 4th year, and 1 to 2 times annually after the 5th year [6]. In our clinical practice, we follow the guidelines for all patients of varied risk with stage 0-III. Our patients had been reminded for FU check via phone or other means (eg. WeChat) as much as possible by nurses. But FU examination and care can only be conducted among patients with good compliance and fail among patients with poor adherence who may require extra attention for post-surgery care due to lack of evidence.

Loss of FU often leads to treatment interruption or treatment plan change which increases the risk of rehospitalization. Besides, as for the breast cancer patients, the annualized hazard of recurrence was the highest during the first 5 years peaking during the first 3 years [7], at which time FU is essential for patient’s long-term prognosis. The post-operation management of breast cancer patients in China includes FU treatment such as endocrine therapy and management of treatment side effects [6]. But at times in our practice, patients’ post-operation care needs, for example, professional guidance in rehabilitation, mental wellbeing, and alternative medicine or Chinese medicine, or lifestyle consultation could not be met. The gap in comprehensive care is calling for continuous multidisciplinary care service for optimal patient management which has been reported beneficial to prognosis [8]. In addition, systemic FU management at the government level in China has not been established as in European and American countries [9, 10]. Therefore, the major challenge in clinical practice in China is that clinicians have no evidence-based protocol regarding follow up meeting patients’ needs and for subgroup patients, for example, for patients with poor accessibility to health facilitates or with comorbidities, or with financial stress. In facing with the increasing individualized FU [11], evidence is urgently required to better define the patient’s care needs and develop appropriate FU program for subgroups to improve the adherence [12]. Currently, FU adherence after breast surgery has not been studied in China.

Therefore, the purpose of this study was to describe the adherence to post-surgery FU guideline and its association with patient’s, tumor and treatment characteristics in a cohort of breast cancer patients having curative surgery in a tertiary hospital in central China.

## Methods

### Data source and study population

Patient registry database in the Breast Surgery Department at Shanxi Bethune Hospital, Shanxi, China, was established in 2012 and collects the information about disease and treatment of all patients with breast cancer admitted in the hospital. Sociodemographic information includes age, medical insurance coverage, marital status, family history. Disease profile includes tumor size, axillary node status, TNM stage, histological subtype, estrogen and progesterone receptor (ER, PR) status, human epidermal growth factor receptor 2 (HER2) status. Detailed information about breast and/or node surgery, chemotherapy, radiotherapy, endocrine therapy, targeted therapy was also included. The study was approved by the Ethics Committee of Shanxi Bethune Hospital (No: YXLL-2019-130).

The database recorded 801 female patients, who were diagnosed with breast cancer and underwent surgical treatment from March 2012 to May 2018. A total of 711 patients were included in the study after excluding 19 patients with stage IV disease, 49 patients with another malignancy, and 22 patients who died before May 2019 (Supplement Figure).

FU was assessed by reviewing records of post-operation imaging examinations including mammography and breast ultrasound [5, 6] at the imaging center of Shanxi Bethune Hospital from April to May 2019. Adherence to FU was defined as having at least one record in the consecutive following year after the surgery, otherwise as loss of FU in the corresponding year.

### Statistical analysis

Annual FU rate was calculated by dividing the number of patients having follow-up examination(s) by the number of eligible patients at the corresponding year (Supplement Table 1) and was compared with Chi-square test.

Considering the recurrence rate peaking in the first 3 years after surgery, patients having had surgery before May 2016 (*n* = 420) were included for subsequent analysis after excluding 291 patients having surgery in 2017 and 2018 (Supplement Figure). FU frequency during the first 3 years after curative surgery was compared by patients, disease, and treatment characteristics using Chi-square test. Factors associated with complete loss FU for all 3 years (loss at years 1, 2, and 3) were investigated with step-wise logistic regressions. In addition, factors associated with loss of FU at year 3 following curative surgery were identified using step-wise generalized linear regression analysis with family being “binomial” and link function being “log” to avoid biased association since the loss of follow up rate was as high as 39.8% at the 3rd year [13]. The adjusted results were the final full model from the regression analyses. The following factors were evaluated: age, medical insurance coverage, family history, marital status, employment, tumor size, axillary nodes status, TNM stage, histological types, ER status, PR, HER2 status, and treatment received. Variables with *P* < 0.10 in univariate analysis were initially included in the multivariate analysis and eliminated each at one time in step-wise regression model justified by likelihood ratio test [13]. The candidate factors included: age, insurance coverage, marital status, tumor size, axillary node surgery, and treatment. Analysis was carried out using SPSS. A two-tailed *p* values < 0.05 were considered as statistically significant.

## Results

### Annual follow-up

The median FU of these patients was 29 months, ranging from 5 to 84 months. The annual FU rates at the first and second year after surgery were 67.1 and 70.6%. There was no significant difference in FU rate between them (*p* = 0.183). However, it dropped to 60.2% (*p* = 0.020)at the third year, and continuously dropped to 51.9% (*p* < 0.001)at the 4th and 43.5% (*p* < 0.001) at the 5th year (Table 1).

**Table 1:** The annual follow-up rate in cancer patients undergone curative surgery during 2012–2018 (*N* = 711)

Annual follow-up	Total patients	Follow-up	Lost follow-up	χ^2^	p*
1st year	711	477(67.1%)	234(32.9%)		
2nd year	551	389(70.6%)	162(29.4%)	1.78	0.18
3rd year	420	253(60.2%)	167(39.8%)	5.42	0.02
4th year	285	148(51.9%)	137(48.1%)	20.00	< 0.01
5th year	177	77(43.5%)	100(56.5%)	33.60	< 0.01

*Annual follow-up rate after surgery were compared with the first year using a Chi-square test

### Follow-up adherence during the first 3 years after surgery

A total of 420 patients was included to assess the FU within 3 years after surgery. The median age at diagnosis was 52 years old (ranging 23–83 years). Among them, five patients (1.2%) had stage 0 cancer, 149 (35.5%) were diagnosed stage I, 202 (48.1%) stage II, 64 (15.2%) stage III. 94 (22.4%) patients had no FU in the first 3 years; while 197 (46.9%) patients had FU imaging each year. Only 7 (1.7%) patients went for FU in the first 2 years but not at the 3rd year.

Loss of FU were more likely in patients who were: older, or having lower insurance coverage, or more extensive axillary node surgery, or no treatments. However, it did not differ by family history, marital or employment status, cancer staging or receptor tests, or breast surgery type (Table 2).

**Table 2:** Post-operation follow-up during the first 3 years by sociodemographic and clinical profile (*N* = 420)

Characteristic	Frequency of follow-up in the first 3 years				Total *N* = 420	p*
	0 ***n*** = 94	1 ***n*** = 50	2 ***n*** = 79	3 ***n*** = 197		
**Age(years)**						< 0.01
< 65	58 (61.7%)	39 (78.0%)	66 (83.5%)	189 (95.9%)	352 (83.8%)	
> 65	36 (38.3%)	11 (22.0%)	13 (16.5%)	8 (4.1%)	68 (16.2%)	
**Medical insurance coverage**						< 0.01
High (> 70%)	16 (17.0%)	10 (20.0%)	24 (30.4%)	77 (39.1%)	127 (30.2%)	
Medium (50–70%)	50 (53.2%)	29 (58.0%)	35 (44.3%)	83 (42.1%)	197 (46.9%)	
Low (< 50%)	28 (29.8%)	11 (22.0%)	20 (25.3%)	37 (18.8%)	96 (22.9%)	
**Family history**						0.89
Negative	88 (93.5%)	47 (94.0%)	76 (96.2%)	186 (94.4%)	397 (94.5%)	
Positive	6 (6.4%)	3 (6.0%)	3 (3.8%)	11 (5.6%)	23 (5.5%)	
**Marital status**						0.31
Unmarried	9 (9.6%)	2 (4.0%)	3 (3.8%)	10 (5.1%)	24 (5.7%)	
Married	85 (90.4%)	48 (96.0%)	76 (96.2%)	187 (94.9%)	396 (94.3%)	
**Employment**						0.84
Employed	65 (69.1%)	37 (74.0%)	51 (64.6%)	130 (66.0%)	283 (67.4%)	
Unemployed	13 (13.8%)	4 (8.0%)	9 (11.4%)	25 (12.7%)	51 (12.1%)	
Retired	16 (17.0%)	9 (18.0%)	19 (24.1%)	42 (21.3%)	86 (20.5%)	
**Tumor size (cm)**						0.40
0–1.9	33 (35.1%)	18 (36.0%)	33 (41.8%)	87 (44.2%)	171 (40.7%)	
2–4.9	54 (57.4%)	30 (60.0%)	41 (51.9%)	105 (53.3%)	230 (54.8%)	
> 5	7 (7.4%)	2 (4.0%)	5 (6.3%)	5 (2.5%)	19 (4.5%)	
**Positive axillary nodal status**						0.80
0	47 (50.0%)	26 (52.0%)	46 (58.2%)	112 (56.9%)	231 (55.0%)	
1–3	29 (30.9%)	18 (36.0%)	20 (25.3%)	61 (31.0%)	128 (30.5%)	
4–9	12 (12.8%)	4 (8.0%)	7 (8.9%)	16 (8.1%)	39 (9.3%)	
> 10	6 (6.4%)	2 (4.0%)	6 (7.6%)	8 (4.1%)	22 (5.2%)	
**TNM stage**						0.86
Stage 0-I	31 (33.0%)	19 (38.0%)	30 (38.0%)	74 (37.6%)	154 (36.7%)	
Stage II	46 (48.9%)	23 (46.0%)	35 (44.3%)	98 (49.7%)	202 (48.1%)	
Stage III	17 (18.1%)	8 (16.0%)	14 (17.7%)	25 (12.7%)	64 (15.2%)	
**Histological subtype**						0.65
Tubular/Mucinous/Papillary	1 (1.1%)	3 (6.0%)	2 (2.5%)	8 (4.1%)	14 (3.3%)	
Ductal/Lobular/Mixed/Metaplastic	90 (95.7%)	46 (92.0%)	76 (96.2%)	186 (94.4%)	398 (94.8%)	
Ductal carcinoma in situ	3 (3.2%)	1 (2.0%)	1 (1.3%)	3 (1.5%)	8 (1.9%)	
**ER status**						0.16
Negative	25 (26.6%)	21 (42.0%)	19 (24.1%)	64 (32.5%)	129 (30.7%)	
Positive	69 (73.4%)	29 (58.0%)	57 (72.2%)	132 (67.0%)	287 (68.3%)	
Missing	0 (0.0%)	0 (0.0%)	3 (3.8%)	1 (0.5%)	4 (1.0%)	
**PR status**						0.28
Negative	31 (33.0%)	24 (48.0%)	25 (31.6%)	73 (37.1%)	153 (36.4%)	
Positive	63 (67.0%)	26 (52.0%)	51 (64.6%)	123 (62.4%)	263 (62.6%)	
Missing	0 (0.0%)	0 (0.0%)	3 (3.8%)	1 (0.5%)	4 (1.0%)	
**HER2 status**						0.41
Negative	77 (81.9%)	38 (76.0%)	65 (82.3%)	152 (77.2%)	332 (79.0%)	
Positive	17 (18.1%)	12 (24.0%)	11 (13.9%)	44 (22.3%)	84 (20.0%)	
Missing	0 (0.0%)	0 (0.0%)	3 (3.8%)	1 (0.5%)	4 (1.0%)	
**Breast surgery**						0.24
Lumpectomy	38 (40.4%)	20 (40.0%)	35 (44.3%)	101 (51.3%)	194 (46.2%)	
Mastectomy	56 (59.6%)	29 (58.0%)	44 (55.7%)	95 (48.2%)	224 (53.3%)	
Missing	0 (0.0%)	1 (2.0%)	0 (0.0%)	1 (0.5%)	2 (0.5%)	
**Axillary surgery**						0.03
Sentinel lymph node biopsy	37 (39.4%)	24 (48.0%)	40 (50.6%)	108 (54.8%)	209 (49.8%)	
Axillary lymph node dissection	50 (53.2%)	24 (48.0%)	36 (45.6%)	88 (44.7%)	198 (47.1%)	
Missing	7 (7.4%)	2 (4.0%)	3 (3.8%)	1 (0.5%)	13 (3.1%)	
**Chemotherapy**						< 0.01
No	47 (50.0%)	12 (24.0%)	12 (15.2%)	28 (14.2%)	99 (23.6%)	
Yes	47 (50.0%)	38 (76.0%)	67 (84.8%)	169 (85.8%)	321 (76.4%)	
**Radiotherapy**						< 0.01
No	62 (66.0%)	23 (46.0%)	25 (31.6%)	39 (19.8%)	149 (35.5%)	
Yes	32 (34.0%)	27 (54.0%)	54 (68.4%)	158 (80.2%)	271 (64.5%)	
**Targeted therapy**						0.03
No	90 (95.7%)	45 (90.0%)	73 (92.4%)	167 (84.8%)	375 (89.3%)	
Yes	4 (4.3%)	5 (10.0%)	6 (7.6%)	30 (15.2%)	45 (10.7%)	
**Endocrine therapy**						< 0.01
No	40 (42.6%)	24 (48.0%)	17 (21.5%)	60 (30.5%)	141 (33.6%)	
Yes	54 (57.4%)	26 (52.0%)	62 (78.5%)	137 (69.5%)	279 (66.4%)	

Abbreviations: *ER* Estrogen receptor status, *PR* Progesterone receptor status, *HER 2* Human epidermal growth factor receptor 2

*p from chi-square test or Fisher’s exact test based on complete cases

Table 3 showed factors associated with complete loss of FU for all 3 years (loss at years 1, 2, and 3) after surgery. Candidate factors eliminated subsequently were tumor size, targeted therapy, marital status, and endocrine therapy. The odds ratio (OR) of loss FU was 2.31 [95% confidence interval (CI) 1.13–4.73] for those aged over 65 years. Patients having lower medical insurance coverage of 50–75% and < 50% were 3.21 (95% CI 1.53–6.73) and 3.58 (95% CI 1.57–8.15) times the odds to loss FU examinations compared to those with high coverage of over 70%. The odds of loss of FU was 2.51 (95% CI 1.39–4.52) times in those with axillary lymph node dissection. Poor FU adherence was more likely in patients without chemotherapy (OR 3.48, 95% CI 1.84–6.57), and radiotherapy (OR 3.90, 95% CI 2.14–7.10). The above significant associated factors with loss of FU at year 3 after surgery were confirmed the same (Supplement Table 2).

**Table 3:** Factors associated with loss of follow-up in the first 3 years after surgery (*N* = 420)

Characteristic	Unadjusted OR (95% CI)	Adjusted OR (95% CI) *
**Age(years)**		
< 65	1.0	1.0
> 65	5.70 (3.28–9.92)	2.31 (1.13–4.73)
**Medical insurance coverage**		
High (> 70%)	1.0	1.0
Medium (50–70%)	2.36 (1.28–4.36)	3.21 (1.53–6.73)
Low (< 50%)	2.86 (1.44–5.66)	3.58 (1.57–8.15)
**Family history**		
Negative	1.0	
Positive	1.24 (0.47–3.24)	
**Marital status**		
Married	1.0	
Unmarried	2.20 (0.93–5.19)	
**Employment status**		
Employed	1.0	
Unemployed	1.15 (0.58–2.28)	
Retired	0.77 (0.42–1.41)	
**Tumor size (cm)**		
0–1.9	1.0	
2–4.9	1.28 (0.79–2.09)	
> 5	2.44 (0.89–6.68)	
**Positive axillary nodal status**		
0	1.0	
1–3	1.15 (0.68–1.94)	
4–9	1.74 (0.82–3.69)	
> 10	1.47 (0.55–3.96)	
**TNM stage**		
Stage 0-I	1.0	
Stage II	1.17 (0.70–1.95)	
Stage III	1.44 (0.73–2.83)	
**Histological subtype**		
In Situ	1.0	
Ductal/Lobular/Mixed/Metaplastic	0.49 (0.11–2.08)	
Tubular/Mucinous/Papillary	0.13 (0.01–1.54)	
**ER**		
Negative	1.0	
Positive	1.32 (0.79–2.20)	
**PR**		
Negative	1.0	
Positive	1.24 (0.76–2.01)	
**HER2**		
Negative	1.0	
Positive	0.84 (0.47–1.52)	
**Surgery of breast**		
Lumpectomy	1.0	
Mastectomy	1.37 (0.86–2.18)	
**Axillary surgery**		
Sentinel lymph node biopsy	1.0	1.0
Axillary lymph node dissection	1.57 (0.97–2.53)	2.51 (1.39–4.52)
**Chemotherapy**		
Yes	1.0	1.0
No	5.27 (3.19–8.70)	3.48 (1.84–6.57)
**Radiotherapy**		
Yes	1.0	1.0
No	5.32 (3.25–8.71)	3.90 (2.14–7.10)
**Targeted therapy**		
Yes	1.0	
No	3.24 (1.13–9.28)	
**Endocrine therapy**		
Yes	1.0	
No	1.65 (1.03–2.64)	

Abbreviations: *ER* Estrogen receptor status, *PR* Progesterone receptor status, *HER 2* Human epidermal growth factor receptor 2

*Adjusted ORs in the final model from step-wise logistic regression analysis by including all the variables in the Table with *P* < 0.10 from univariate logistic regression and eliminate each at a time justified by likelihood ratio test [13]

## Discussion

This study showed that FU rates after surgery decreased within the five-year period from 67.1% in the first year to 60.2% at the third year, down to 43.5% at the fifth year. This decreasing trend has been consistently reported in other populations [14–16]. For example, a Dutch study of patients with breast cancer found that the first year of FU was 82% and it dropped to 68.5% at the fourth year [14]. A Canadian longitudinal study found that about 80% of patients had at least one FU at the first 4 years, and it dropped to 73% at the fifth year [15]. An American study on outpatient FU rate showed a decrease from 50% at the first year to 27% at the third year [16]. Although the annual FU rates from the existing studies vary substantially, the annual down trend is unquestionable. The FU rates in this study was generally lower than that of European and American countries. One reason is that in China, especially in the central region, primary care or a systematic tracking data platform at the provincial or city level has not been fully established or well functioned [17]. In addition, targeted procedures to promote post-surgery FU in subgroups was unable to be implemented due to missing evidence. This study provided some evidence to promote FU among subgroups. Further investigations on the feasibility of implementing routinely collected PROM (patient reported outcome indicators) and its impact on improving FU care of breast cancer patients in China is needed [18], as systematic review of 34 studies mostly among Caucasians reported PROM collection in routine breast cancer care is feasible and has promising results [19].

We found that elderly breast cancer patients were prone to loss of FU, which was consistent with other studies [14, 16, 20, 21]. Older patients are more likely diagnosed with other comorbidities such as hypertension and diabetes and frail to have a FU visit. In addition, elder patients have lower level of education as showed from the China’s national data, which limit patients’ understanding of the disease and its treatment regimens, and the importance of post-surgery FU [22]. On the other hand, it has been confirmed that patients can benefit from education and counselling, which has positive impact on medication adherence [23].

We found among this group of patients in central China, poor FU adherence was significantly associated with lower insurance coverage, possibly due to financial stress. Cancer-related “financial toxicity” [24] is a major obstacle for patients to have FU examination or to continue treatment after surgery as indicated from our study in patients with breast cancer from the same hospital [25]. An American survey of patients with early-stage breast cancer reported that 77% of patients had financial burden related to the disease [26], similar to another American study indicating that 44% of breast cancer patients had at least moderate economic difficulties, and up to 88% were concerned about treatment-related costs [27]. Clinicians should take this into consideration and discuss with patients for informed decision to improve FU for better clinical outcome. In addition, policy support, especially for patients with high recurrence risk, should be developed by incorporating necessary FU examinations into the medical insurance system.

This study shows that extensive axillary surgery was associated with the increased risk of loss of FU. Theoretically speaking, patients with the extensive surgery are more prone to lymphedema requiring close FU. Rather, the previous researches have consistently found that patients with more serious disease are more likely to give up disease management [28, 29]. These results indicated the needs for clinicians to attempt psychological consultation or education during the FU for patients to foster a positive attitude of their current physical condition and to alleviate or eliminate the negative emotions including fear and anxiety [30]. It could facilitate patients’ understanding of the condition and the treatment and active participation in decision-making.

We found that patients having had radiotherapy or chemotherapy were more likely to FU examinations after surgery. This result was consistent with the reports by Enright and Neuman that breast cancer patients who have undergone more intensive treatment have better adherence and more frequent FU [20, 31]. Generally speaking, patients with more severe disease should be treated with more intensive treatment. This suggests that the association with FU adherence of intensive treatment cannot be explained by severity of disease alone but related to treatment adherence. For example, it is reported that adherence with endocrine therapy fell from 90% in the first year to 50% at the fifth year [32]. The same decreased trend was also observed in our patients. Further study is needed to prove the relationship between the follow-up and treatment adherence.

Limitations of the study should be noted. Firstly, the loss of FU rate may be overestimated considering that some patients may go to other local hospitals for the post-surgery FU due to various reasons such as transportation. Secondly, the loss of FU in the first 3 years was investigated and the evaluation of long-term FU assessment was not available due to the limited case numbers. Lastly, the impact of adherence to FU examination on survival warrants further assessment.

Despite those limitations, we were able to identify subgroup among this cohort of breast patients with higher probability of loss post-operation FU. This could help health care staff to make more efforts to track the subgroups and to apply more rigorous post-surgery patient care. In addition, there is a need at the government level to establish a high-level database to comprehensively and accurately track the care and the survival of patients after surgery.

## Conclusions

To sum up, we found that the annual FU rate after curative surgery for breast cancer survivors decreased significantly within 5 years, and that the probability of poor adherence to FU examinations was significantly higher in patients aged over 65 years with lower medical insurance coverage, having extensive axillary lymph node dissection, and no radiotherapy or chemotherapy. These results suggested the need for improving adherence to FU in higher-risk subgroups and help clinicians to develop tailored patient management after curative surgery.

## Supplementary Information

**Additional file 1: Table S1.** Annual follow-up among 711 patients during 2012–2019. **Table S2.** Factors associated with loss of follow-up at the 3rd year after surgery (*N* = 420). **Figure S1.** Patients included in the analyses during 2012–2018.

## Data Availability

All data generated or analyzed during this study are included in this published article and its supplementary information files. The original data are available upon request to the corresponding author.

## Abbreviations

NCCN: National Comprehensive Cancer Network
ER: Estrogen receptor
PR: Progesterone receptor
HER 2: Human epidermal growth factor receptor 2
OR: Odds ratio
95% CI: 95% confidence interval
