# Supplementary material for: Adherence to post-surgery follow-up assessment and its association with sociodemographic and disease characteristics in patients with breast cancer in Central China
Source: BMC Cancer. 2020 Nov 12;20:1098. doi: 10.1186/s12885-020-07600-y (PMC7659108; doi:10.1186/s12885-020-07600-y)
Supplement: Supplementary file 1 — Additional file 1: Table S1. Annual follow-up among 711 patients during 2012–2019. Table S2. Factors associated with loss of follow-up at the 3rd year after surgery (N = 420). Figure S1. Patients included in the analyses during 2012–2018. [file 12885_2020_7600_MOESM1_ESM.doc]

Supplement Table 1 Annual follow-up among 711 patients during 2012-2019

| Year of surgery | Followed | Follow up year | | | | | | | |
| --- | --- | --- | --- | --- | --- | --- | --- | --- | --- |
|  |  | 2013 | 2014 | 2015 | 2016 | 2017 | 2018 | 2019 | All patients of each year |
| 2012 | Yes | 2 | 12 | 11 | 10 | 6 | - | - | 16 |
|  | No | 14 | 4 | 5 | 6 | 10 |  |  |  |
| 2013 | Yes | - | 43 | 53 | 47 | 47 | 41 | - | 83 |
|  | No |  | 40 | 30 | 36 | 36 | 42 |  |  |
| 2014 | Yes | - | - | 47 | 44 | 40 | 34 | 30 | 78 |
|  | No |  |  | 31 | 34 | 38 | 44 | 48 |  |
| 2015 | Yes | - | - | - | 69 | 70 | 58 | 57 | 108 |
|  | No |  |  |  | 39 | 38 | 50 | 51 |  |
| 2016 | Yes | - | - | - | - | 101 | 105 | 97 | 135 |
|  | N |  |  |  |  | 34 | 30 | 38 |  |
| 2017 | Yes | - | - | - | - | - | 101 | 105 | 131 |
|  | No |  |  |  |  |  | 30 | 26 |  |
| 2018 | Yes | - | - | - | - | - | - | 114 | 160 |
|  |  |  |  |  |  |  |  | 46 |  |

Follow up rate at the 1st year=(2+43+47+69+101+101+114)/(16+83+78+108+135+131+160)=67.1%

Cases at 1 year after surgery =16+83+78+108+135+131+160=711

Follow up rate at the 2nd year=(12+53+44+70+105+105)/(16+83+78+108+135+131)=70.6%

Cases at 2 years after surgery=16+83+78+108+135+131=551

Follow up rate at the 3rd year=(11+47+40+58+97)/(16+83+78+108+135)=60.2%

Cases at 3 years after surgery=16+83+78+108+135=420

Follow up rate at the 4th year=(10+47+34+57)/(16+83+78+108)=51.9%

Cases at 4 years after surgery=16+83+78+108=285

Follow up rate at the 5th year=(6+41+30)/(16+83+78)=43.5%

Cases at 5 years after surgery=16+83+78=177

Supplement Table 2 Factors associated with loss of follow-up at the 3^rd^ year after surgery (N=420)

| **Characteristic** | **Unadjusted OR (95% CI)** | **Adjusted OR (95% CI)*** |
| --- | --- | --- |
| **Age（years）** |  |  |
| <65 | 1.0 | 1.0 |
| >65 | 5.58 (3.12-10.00) | 3.20 (1.56-6.53) |
| **Medical insurance coverage** |  |  |
| High (>70%) | 1.0 | 1.0 |
| Medium (50-70%) | 3.15 (1.91-5.19) | 3.83 (2.13-6.91) |
| Low (<50%) | 2.74 (1.54-4.88) | 3.13 (1.60-6.16) |
| **Family history** |  |  |
| Negative | 1.0 |  |
| Positive | 1.18 (0.50-2.75) |  |
| **Marital status** |  |  |
| Unmarried | 1.0 |  |
| Married | 1.86 (0.81-4.25) |  |
| **Employment status** |  |  |
| Employed | 1.0 |  |
| Unemployed | 0.78 (0.39-1.62) |  |
| Retirement | 0.11 (0.60-2.04) |  |
| **Tumor size (cm)** |  |  |
| 0-1.9 | 1.0 |  |
| 2-4.9 | 1.45 (0.96-2.18) |  |
| >=5 | 2.68 (1.02-7.03) |  |
| **Positive axillary nodal status** |  |  |
| 0 | 1.0 |  |
| 1-3 | 1.25 (0.81-1.95) |  |
| 4-9 | 1.20 (0.60-2.39) |  |
| >10 | 2.06 (0.85-4.97) |  |
| **TNM stage** |  |  |
| Stage 0-I | 1.0 |  |
| Stage II | 1.20 (0.78-1.84) |  |
| Stage III | 1.45 (0.80-2.62) |  |
| **Histological subtype** |  |  |
| In Situ | 1.0 |  |
| Ductal/Lobular/Mixed/Metaplastic | 0.27 (0.04-1.80) |  |
| Tublar/Mucinous/Papillary | 0.67 (0.16-2.73) |  |
| **ER** |  |  |
| Negative | 1.0 |  |
| Positive | 1.18 (0.77-1.81) |  |
| **PR** |  |  |
| Negative | 1.0 |  |
| Positive | 1.19 (0.79-1.80) |  |
| **HER2** |  |  |
| Negative | 1.0 |  |
| Positive | 1.17 (0.72-1.92) |  |
| **Surgery of breast** |  |  |
| Lumpectomy | 1.0 |  |
| Mastectomy | 1.44 (0.97-1.92) |  |
| **Axillary surgery** |  |  |
| Sentinel lymph node biopsy | 1.0 | 1.0 |
| Axillary lymph node dissection | 1.56 (1.04-2.33) | 1.94 (1.20-3.12) |
| **Chemotherapy** |  |  |
| Yes | 1.0 | 1.0 |
| No | 3.26 (2.04-5.19) | 2.11 (1.18-3.79) |
| **Radiotherapy** |  |  |
| Yes | 1.0 | 1.0 |
| No | 3.51 (2.31-5.33) | 2.58 (1.55-4.28) |
| **Targeted therapy** |  |  |
| Yes | 1.0 |  |
| No | 2.20 (1.08-4.48) |  |
| **Endocrine therapy** |  |  |
| Yes | 1.0 |  |
| No | 1.42 (0.94-2.14) |  |

Abbreviations: ER-estrogen receptor status; PR-progesterone receptor status; HER 2-human epidermal growth factor receptor 2 status.

*Adjusted OR in the final model from step-wise Generalized linear regression analysis by including all the variables in the Table (P<0.10) from univariate analysis


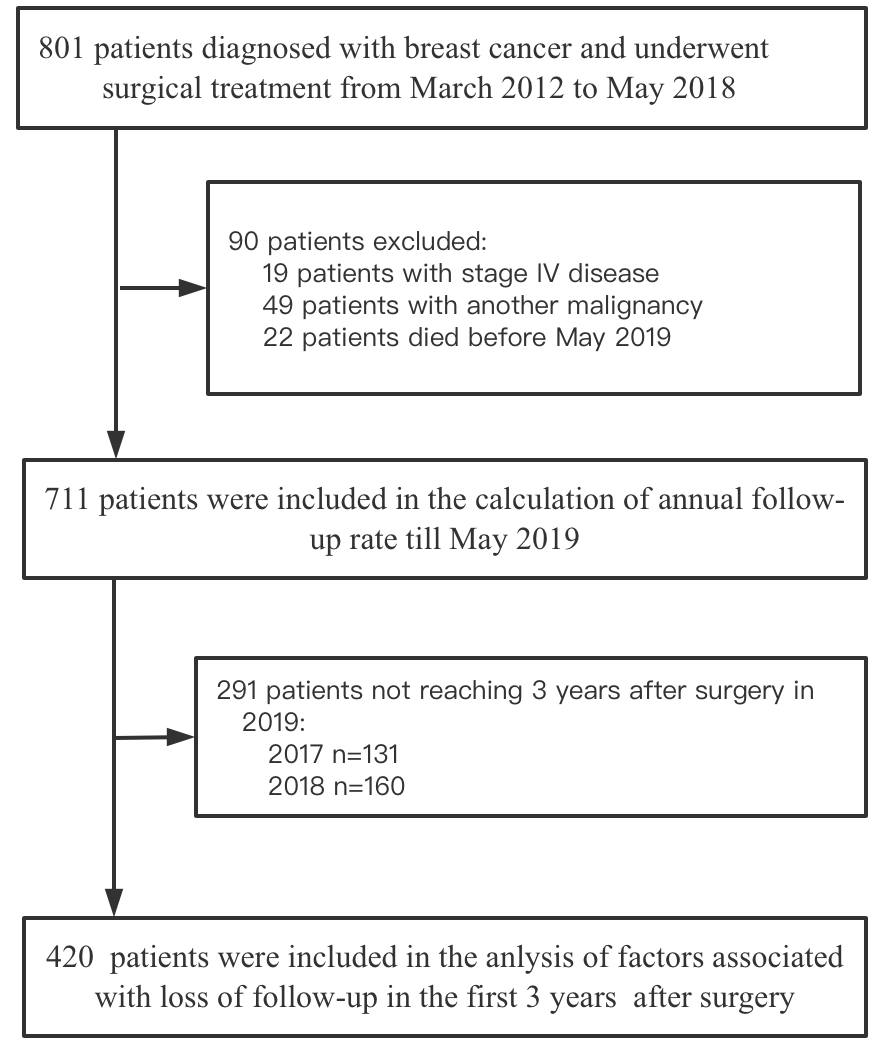


Supplement Figure. Patients included in the analyses during 2012-2018
